# Opposing needling for analgesia and rehabilitation after unilateral total knee arthroplasty: a randomized, sham-controlled trial protocol

**DOI:** 10.1186/s13063-020-04251-z

**Published:** 2020-05-07

**Authors:** Hai Huang, Xiuling Song, Ling Zhao, Lin Zheng, Lianbo Xiao, Yuelai Chen

**Affiliations:** 1 Guanghua Hospital, Shanghai University of Traditional Chinese Medicine, 540 Xinhua Rd., Shanghai, 200052 China; 2 Shanghai University of Traditional Chinese Medicine, 1200 Cailun Rd., Shanghai, 201203 China

**Keywords:** Opposing needling, Total knee arthroplasty, Sham acupuncture, Pain, Protocol

## Abstract

**Background:**

This randomized controlled clinical trial aims to evaluate the efficacy and safety of opposing needling in patients undergoing unilateral total knee arthroplasty (TKA). Opposing needling is one of the special needling methods used in acupuncture and moxibustion therapy. It involves needling acupoints on the contralateral side for pain management. Although, opposing needling is used for pain management in clinics, evidence to support its effectiveness as an analgesic after total knee arthroplasty is scant. We designed a randomized controlled clinical trial to evaluate efficacy and safety of opposing electroacupuncture (EA) in alleviating pain associated with unilateral total knee arthroplasty.

**Methods/design:**

This is a protocol for a randomized controlled patient- and assessor-blinded trial with three parallel arms (A, opposing EA; B, operated side EA; C, sham EA). *Yinlingquan* (SP9), *Yanglingquan* (GB34), *Futu* (ST32), and *Zusanli* (ST36) acupoints are selected for all three groups. In group A, the healthy side will be treated with EA, while the operated side will be administered sham EA. In group B, the operated side will be treated with EA while on the healthy side sham EA will be used. For group C, sham EA will be used on both sides. All patients in the three groups will receive treatment once a day for 3 days. The post-operative pain measured using a visual analogue scale score (including pain while resting and being active) and the additional dose of the patient-controlled analgesic pump after operation will be recorded as the primary outcomes. Secondary outcomes such as knee function and swelling, range of motion (including active and passive range of motion), post-operative anxiety, and acupuncture tolerance will also be assessed.

**Discussion:**

Opposing needling is a potential non-pharmacological treatment for relieving pain and improving functional rehabilitation after TKA, during which patients receive acupuncture on the healthy side rather than on the operated side. This sham controlled clinical trial, designed to evaluate efficacy and safety of opposing needling for patients after TKA, will provide evidence for pain management and functional rehabilitation after unilateral TKA.

**Trial registration:**

ChiCTR, ChiCTR1800020297 (http://www.chictr.org.cn/edit.aspx?pid=34231&htm=4). Registered 22 December 2018.

## Background

Total knee arthroplasty (TKA) is one of the best treatments for advanced knee osteoarthritis (KOA) [1], providing a standardized treatment for relieving pain, remodeling knee function, improving quality of life, and improving postural balance [2, 3]. Although a number of benefits are associated with TKA, it is imperative to address the potentially increased rate of complications of post-operative pain, post-operative infection, and deep vein thrombosis among these older patients [4]. The management of post-operative pain is a recurring problem that needs effective rectification. Post-operative pain still accounts for 14.1% of the most common reasons for re-admission within 90 days of surgery as reported in a European study [5]. Thus, post-operative pain is still a major complication that arises after TKA [6]. A number of patients are dissatisfied with the efficacy of commonly used analgesics post-operation and the ancillary side effects that come with them [7, 8]. Kim et al. have focused on the risk factors associated with persistent chronic opioid usage after surgery and suggest that targeted opioid reduction programs may be appropriately implemented to manage this high-risk population [9]. Effective and adequate pain management can restore joint function, prevent chronic pain [10], and allow for a more comfortable and rapid rehabilitation [11].

Acupuncture from ancient Chinese medicine is a safe treatment method without serious side effects [12]. It can be used as a complementary and alternative therapy for many diseases such as dyspepsia [13], overactive bladder [14], stress urinary incontinence [15], etc. Clinically, it can also be used as a drug-free intervention to relieve post-operative pain and help reduce the use of opioid analgesics [16]. Post-operative pain control following TKA using acupuncture therapies has more potential to develop into effective treatment regimens than other alternative therapies [17]. Tedesco et al. [16] systematically evaluates and meta-analyses the drug-free interventions available as post-operative analgesia in the context of TKA. Medium-quality evidence shows that electrotherapy and acupuncture after TKA can reduce the use of opioids and delay opioid consumption. Electroacupuncture (EA) can stimulate acupoints to alleviate pain by linking up a painless weak electrical stimulation. EA not only relieves acute pain [18] but is also effective against chronic pain [19]. This may, therefore, be considered as an adjunct intervention for long-term pain relief in standard non-drug therapy. In Mikashima et al.’s study [20], acupuncture on the affected side provided an effective treatment for post-operative acute pain. And in our previous trial [21] on applying EA for post-operative pain management after TKA, the technique of sham EA was mastered. During our study, however, the researchers were concerned that acupuncture near the incision may increase the risk of post-operative infection, so we considered the treatment of opposing needling after operation.

Opposing needling, also known as healthy side acupuncture or contralateral acupuncture, is one of the nine needling methods of acupuncture first recorded in *Huangdi Neijing*. According to the patient’s condition, the needling method is used at select acupoints on the right side of the body when the left side is affected, and vice versa [22, 23]. Opposing needling therapy is used in various types of neuralgia and functional rehabilitation therapies in China [24, 25], wherein acupoints are selected generally on the healthy side corresponding to the affected side [26]. Studies [27, 28] have indicated distinct anti-nociceptive effects and mechanisms between ipsilateral and contralateral acupuncture and moxibustion. And anterior cingulate cortex plays an important role in contralateral acupuncture and moxibustion. Alleviation of pain is one of the major areas for the use of opposing needling; however, it has not been used for the treatment of post-operative pain.

Thus, the study protocol that we designed is a clinical study on the opposing needling method for analgesia and rehabilitation after unilateral total knee arthroplasty.

## Methods/design

### Objective

The aim of this study is to investigate the effectiveness of opposing EA compared with operated side EA or sham EA treatment in patients after unilateral TKA, and to provide clinical evidence for the treatment of post-operative pain by opposing needling.

### Study design

**Fig. 1:**
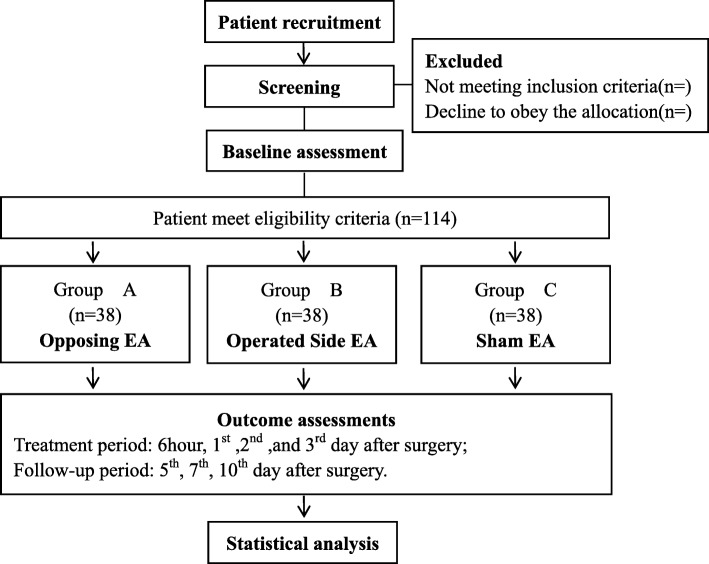
The flow diagram is intended to depict the passage of participants through this RCT

A randomized, controlled, three-arm, patient- and assessor-blinded trial will be conducted to compare the efficacy of two true EA groups (opposing EA and operated side EA) with a sham EA group (Fig. 1).

The study will be sequentially conducted as follows: 114 patients in Guanghua Hospital, Shanghai University of Traditional Chinese Medicine with osteoarthritis are included (surgical contraindications are excluded); after admission, unilateral TKA will be performed as scheduled. A day before the surgery, the eligible patients will be assessed at baseline and their consent will be obtained in writing. Patients will then be randomly assigned (on a 1:1:1 ratio) to the opposing EA group, the operated side EA group, or the sham EA group. Random numbers will be generated by the SPSS 22.0 software and sealed in opaque envelopes. Only the operator in charge of acupuncture treatment is authorized to open the envelope to obtain the group code.

The three groups of subjects will receive interventions on three consecutive days after surgery, once every day, by a clinician with more than 3 years of experience in acupuncture intervention. The participants will be blinded to the intervention measures. The success of the visit would be evaluated during the last visit of each participant. Researchers who did not participate in the treatment and who did not know the allocation results will perform the outcome assessment. Unblinding before the completion of the study will only be allowed in the case of medical emergencies or severe adverse events (SAEs).

All patients will receive the same post-operative analgesic dose with a fentanyl patient-controlled analgesic (PCA) pump (continuous infusion rate 0.25 μg/(kg·h)). Additional analgesics will be provided as per each patient’s requirements ascertained by the visual analogue scale (VAS) score > 6, and the use of analgesics will be recorded in their case report form (CRF).

### Eligibility

#### Inclusion criteria

Subjects are eligible to participate if they meet the following criteria: (1) aged 40 to 75 years; (2) meet the Clinical Classification Criteria for Osteoarthritis of the Knee as recommended by the American College of Rheumatology; (3) undergoing unilateral TKA under general anesthesia during hospitalization without surgical contraindications; (4) American Society of Anesthesiologists (ASA) grade I or II [29].

#### Exclusion criteria

Participants will be excluded if they are experiencing or have a history of the following: (1) acupuncture cannot be performed because of skin lesions at acupoints; (2) lower extremity sensory disturbance or abnormality; (3) severe arrhythmias, heart failure, chronic obstructive pulmonary disease, epilepsy, mental disorders; (4) received acupuncture treatment in the past 1 month.

#### Dropout criteria

Any participant will be dropped from the study if she or he (1) cannot tolerate acupuncture after operation or cannot complete the study protocol scheduled; (2) cannot continue the acupuncture procedure because of surgical factors; (3) refuses to receive EA or sham EA treatment; (4) shows SAEs; (5) violates the protocol or refuses to follow up.

### Treatment protocol

**Fig. 2:**
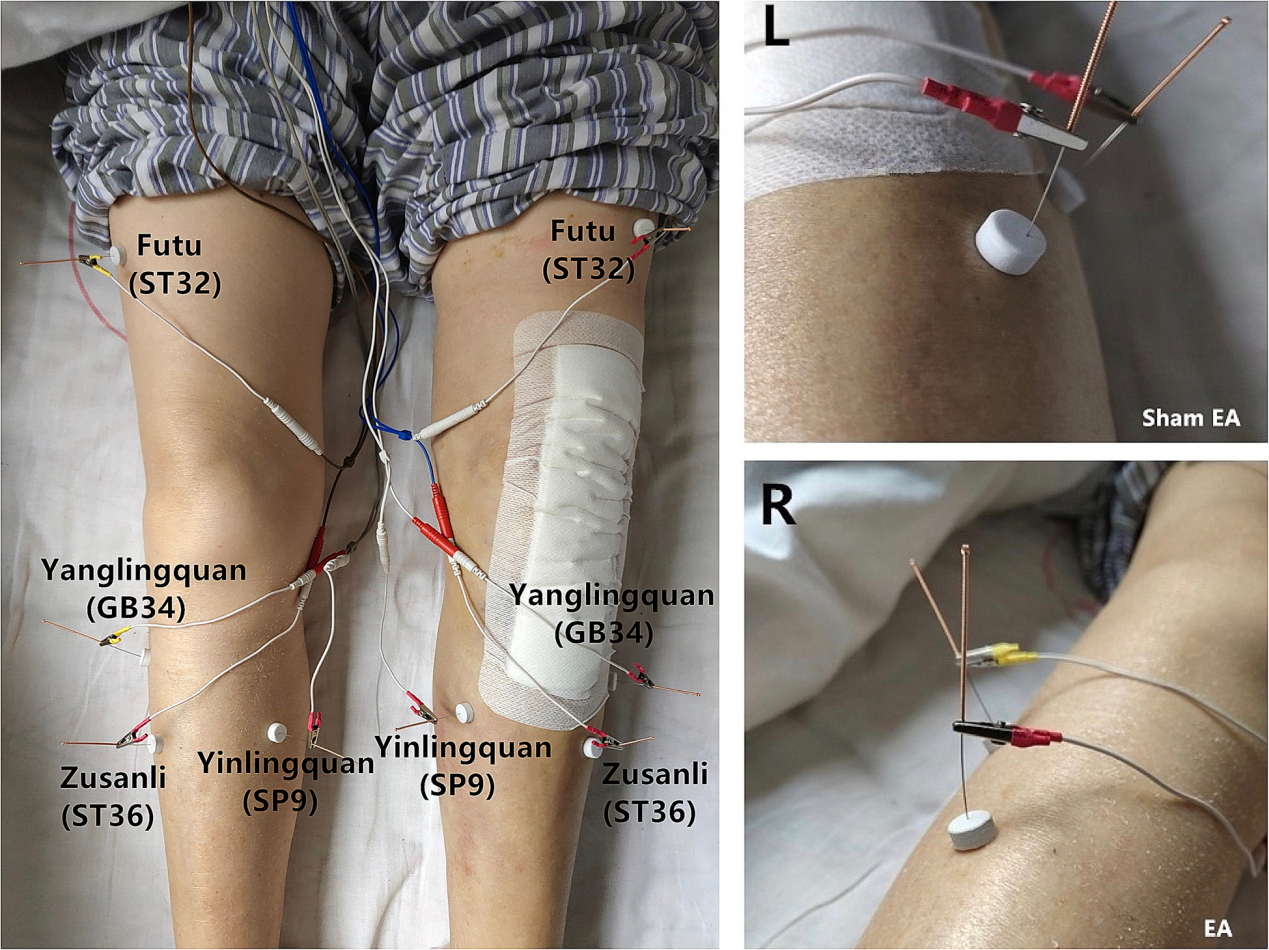
Acupoints of treatment, EA, and sham EA

On the basis of the records in the ancient Traditional Chinese Medicine work *Huangdi Neijing*, treatment for flaccidity is aimed at the *yangming* meridian (ST). Acupoints in the stomach channel of foot-*Yangming* are the most involved ones, featuring a distribution around knees. *Futu* (ST32) and *Zusanli* (ST36) points on the stomach meridian of foot-*Yangming* are selected. Furthermore, according to the meridians and acupoints with opposite locations, combined with the principle of local acupoint selection [30], *Yinlingquan* (SP9) and *Yanglingquan* (GB34) are selected as a pair of acupoints located, respectively, at yin meridians and yang meridians with opposite locations; it can regulate yin and yang, qi, and blood with the selection of lesser points but with a better effect [22]. After discussion and consensus among acupuncture experts, the above four acupoints have been chosen for the study (Fig. 2).

**Fig. 3:**
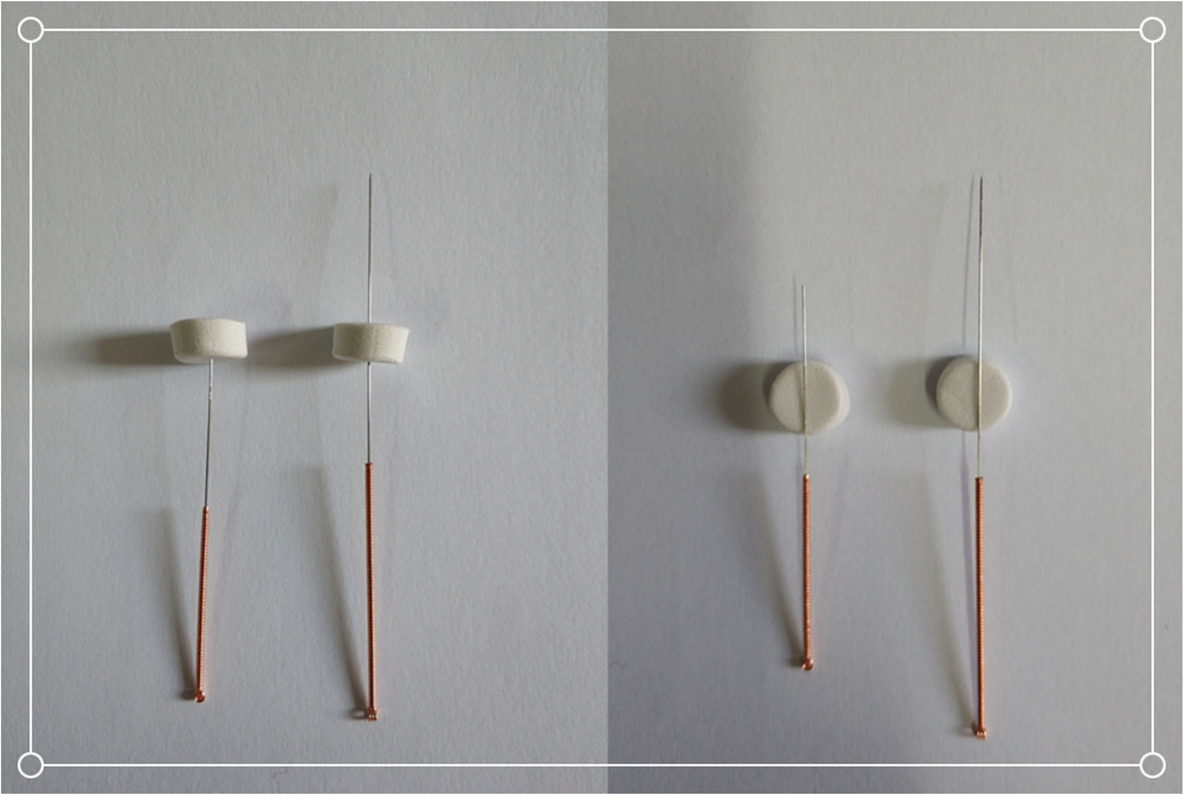
Needle and sham needle

Patients will stay in a relaxed and supine position before the onset of treatment. The acupuncturist will untie the elastic bandage that was fixed during the operation, so that the acupoints are exposed. The skin around the acupoints is sterilized with cotton dipped in 75% alcohol, and a sterile adhesive pad (10 mm in diameter, 5 mm thick) is pasted on each of the acupoints (Fig. 3). The sterile adhesive pad is made of a special sponge (Suzhou Medical Appliance Factory, China) with one sticky side which can be pasted on the skin. The role of the sterile rubber pad is to fix the location of acupoints and to ensure subject blinding. The participants in the three groups will receive a 30-min treatment on the first, second, and third day after operation by the same acupuncturist, three times per session. It will be difficult for them to ascertain which group they belong to.

#### Opposing EA group

The healthy side of the lower limbs will be treated with true EA while on the operated side sham EA will be used.

#### Operated side EA group

The operated side of the lower limbs will be treated with true EA while the healthy side will be treated with sham EA.

#### Sham EA group

Sham EA will be used on both the operated and healthy side.

The specific procedures of EA and sham EA are as follows (Fig. 2):

##### Electroacupuncture (EA)

Needles (0.30 mm in diameter, 40 mm in length, Hwato Brand, Suzhou Medical Appliance Factory, China) will be inserted vertically into the acupoints through the adhesive pads to a depth of 25–30 mm (Fig. 3). Acupuncturists will twist to achieve and enhance the sensation of needles inserted into the skin around acupoints, such as numbness, heaviness, aches, etc. (known as *De Qi*). Subsequent electrical stimulation (SDZ-V electroacupuncture apparatus, Huatuo brand, Suzhou Medical Appliance Factory, China) will be connected to each needle handle (a connecting wire connects a pair of acupoints, GB34 to ST32 and ST36 to SP9) and a continuous wave of 2 Hz frequency and an intensity of 1 mA will be maintained.

##### Sham electroacupuncture (Sham EA)

The blunt needle (Fig. 3, 0.30 mm in diameter, 25 mm in length, Hwato Brand, Suzhou Medical Appliance Factory, China) will reach the skin through the adhesive pads without penetrating the skin. The needle handle is rotated three times to simulate the real acupuncture procedure. The sham electrical stimulation apparatus looks like the real one, but with the inner metal wire cut off so no current passes through the connecting wire between the two needle handle (GB34 to ST32 and ST36 to SP9); a continuous wave of 2 Hz frequency and an intensity of 1 mA is applied [31].

During the treatment, the needle handles are rotated on both sides three times per 5 min by acupuncturist.

### Outcome measures

#### Primary outcome measurement

The main evaluation criterion is pain, which is divided into two parts.

The first part is a visual analogue scale (VAS). The assessment schedule is shown in Table 1. Independent assessors who are not involved in acupuncture treatments will perform the VAS assessments (asking subjects to indicate the intensity of pain at the most painful points while resting and walking). The 0–100 mm VAS is used to evaluate pain intensity, wherein 0 mm denotes painless and 100 mm represents the most intolerable pain [32]. It allows patients to mark their own feelings on the scale as the evaluation score of pain. In addition to evaluating resting pain at 6 h after the operation, the pain in both the resting and active state is measured immediately after treatment on the first to third days after the operation, and assessed at follow-up on the fifth, seventh, and tenth days after the operation.

**Table 1:** Schedule of trial enrollment, interventions, and assessments

Items	Enrollment (pre-operative)	Allocation (baseline)	Treatment (post-operative)				Follow-up		
Time point (hour/day)	-1	-1	6 h	1d	2d	3d	5d	7d	10d
Treatment sessions (n)	–	–		1	2	3			
Informed consent	√								
Assessment of eligibility	√								
VAS (resting)		√	√	√	√	√	√	√	√
VAS (active)		√		√	√	√	√	√	√
Additional dose released by PCA pump					√				
HSS score		√				√			√
HAMA score		√				√			√
AROM		√				√	√	√	√
PROM		√				√	√	√	√
COK				√		√	√	√	√
Discomfort and acceptance of EA				√	√	√			
Assessment of blinding method						√			
Postoperative complications and adverse events				√	√	√			

*VAS* Visual Analogue Score, *PCA* patient controlled analgesia, *HSS* hospital for special surgery, *HAMA* Hamilton Anxiety Scale, *COK* circumference of knee, *AROM* active range of motion, *PROM* passive range of motion

The second part is to record any additional doses from the patient-controlled analgesic pump after the operation, and to evaluate it uniformly on the second day after the operation (at 24 h after the first treatment).

#### Secondary outcome measurements

The secondary outcome measurements are mainly related to rehabilitation after the TKA operation. They include the following.

The knee score of patients will be measured at three time points—the day before operation and on the third and tenth days after the operation—using the Hospital for Special Surgery Knee Score (HSS), commonly used in similar studies. The HSS gives a maximum of 100 points and includes pain (30 points), function (22 points), range of motion (18 points), muscular strength (10 points), deformity (10 points), instability (10 points), and subtractions (three items) [33, 34].

Active and passive range of motion (ROM) of knee joint will be measured by a standard joint protractor measure [35] on the day before the operation and the third, fifth, seventh, and tenth days after the operation.

Based on the ratio of knee circumference on the first day after the operation, the improvement of swelling around the knee joint will be evaluated on the third, fifth, seventh, and tenth days after the operation according to the following formula: (COK on the Xth-COK on the first day after operation) /(COK on first day after operation), where COK is the circumference of the knee at suprapatellar pole20 [20].

The Hamilton Anxiety Scale (HAMA) consists of 14 items, each of which has five levels [36]. In this study, the severity of anxiety in patients will be assessed on the day before the operation, and the third and tenth days after the operation.

### Assessment of the subject blinding success rate for acupuncture

Patients of the three groups will be asked to guess their assigned group to assess the masking effectiveness of the trial (Table 2), at 5 min after the end of the third treatment. Subjects will be asked the following questions: (1) How does the needle sensation feel (sensations induced by acupuncture, like sourness, numbness, distending, heaviness, etc.)? Please ring a number between 0 (no needle sensation) and 10 (unbearable needle sensation). (2) Have you ever received acupuncture? (3) Have you ever received electroacupuncture? (4) Do you think you have been treated at acupoints? (5) Are you sure you are receiving acupuncture treatment? [37] (6) At which lower extremity do you think you have been needled?

**Table 2:**
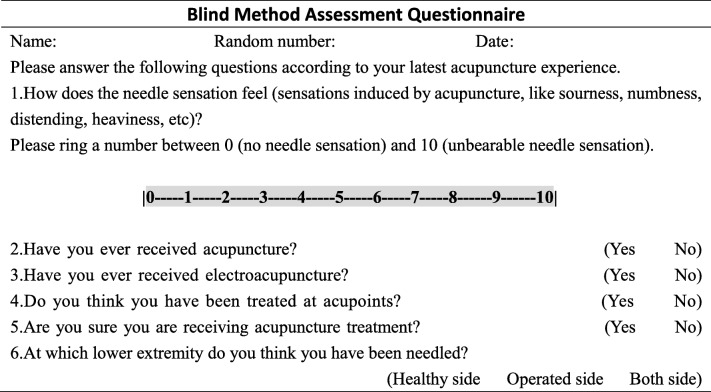
Blind method assessment questionnaire

Note: The questionnaire survey will be completed within 5 min after the end of the third treatment

### EA safety and adverse events

Although acupuncture has been deemed a safe and reliable therapy, its adverse effects and complications associated should raise concerns [12]. Clinically, the incidence of minor adverse events related to acupuncture may be higher, such as pain, local infection, fatigue, local hematoma, subcutaneous hemorrhage, etc. [38, 39]. Some of these might be avoidable [40]. During the treatment period, acupuncturists will strictly abide by the operating rules of electroacupuncture [41]. All acupoints are around the knee joint(s). Acupuncture insertion is in the superficial or subcutaneous regions. Occasionally, there will be a small amount of bleeding or bruising. The bleeding can be arrested by pressing with sterile cotton swabs or cotton balls. The bruising usually disappears within 2–3 days [32].

In this study, during the entire clinical trial, observers who are also blinded to group allocation will record the adverse events (AEs) related to acupuncture treatment in detail and enumerate the incidences. AEs will be managed by acupuncturists and relevant clinical experts within 24 h. Any adverse event, whether related to treatment or not, is reported by the participants and practitioners at every visit.

### Ethics and dissemination

The protocol of this clinical trial adheres to the SPIRIT guidelines. Willingness to be randomly assigned and understanding and signing the informed consent is imperative for every participant in the study. All study participants will agree in writing, and researchers will provide information on this trial in detail to each person. The ethical validity of the study has been assessed and approved by the Institutional Review Board of Guanghua Hospital, Shanghai University of Traditional Chinese Medicine (No.2018-K − 18). The study is registered in the Chinese Clinical Trials Registry (trial registration number ChiCTR1800020297), where the website will present the result of the study.

### Data management

In this study, YLC and LBX, with at least 20 years of clinical experience, are the initiators and instructors. HH is the acupuncturist for the study, ^4^LZ will be the clinical assessor, and ^3^LZ and XLS will conduct data management and analyze results. Any changes to the protocol will be approved by the ethics committee. Due to the small sample size and the known low risk of acupuncture [38], a formal data monitoring committee will not be necessary. During the trial, independent investigators in the hospital will regularly monitor and audit the data collected to ensure good data quality. Within one week after completing the data collection, all data will be entered into a password-protected computer by two independent trained research assistants. Finally, CRFs and research documents will be filed in the department research cabinet.

### Statistical analysis

The data in this study will be analyzed using the Statistical Package for Social Science software (SPSS version 22.0, IBM Corp, New York). After data collection, statistical analysis will be performed by a special statistician. Data analysis will be based on the intention-to-treat (ITT) principle [42], considering significance levels < 0.05 as statistically significant.

Continuous data will be represented by mean, standard deviation, median, and interquartile ranges and the categorical data by percentages.

According to whether the data are normally distributed, the analysis of baseline will be performed using a *t*-test or a Wilcoxon rank sum test for continuous data and a chi-squared test or Fisher’s exact test for categorical data. Comparisons among the three groups will be analyzed by one-way analysis of variance (ANOVA) [43]. When the distribution is not normal, nonparametric tests will be used. Thus, the primary outcome will be assessed using a one-way, repeated-measures ANOVA to test the influence of the time, the group, and the time*group interaction on pain. Comparisons of categorical data among groups will be tested by the chi-square test or the Mann Whitney U-test.

### Sample size

According to the data from the previous preliminary study, through observing the analgesic effect of opposing EA three times after TKA operation, the average VAS score was 37.0 with a standard deviation = 22.6, the average value of sham EA was 46.9, and the standard deviation was 12.5, with a significance level α = 0.05 (two-sided) and power (1 − β) = 0.8. According to the following sample size formula [44, 45], μ is the mean and σ is the standard deviation, κ = n_A_/n_B_ is the matching ratio. τ is the number of comparisons to be made. Look-up table Ζ can be obtained. The sample size was calculated as 96 cases; taking a 15% dropout rate into account, 38 subjects are required in each of the three groups.

$$ {n}_A=\left({\sigma}_A^2+{\sigma}_B^2/\upkappa \right){\left(\frac{Z_{1-\alpha /\tau }+{Z}_{1-\beta }}{\mu_A-{\mu}_B}\right)}^2 $$

## Discussion

In recent years, with the development of fast-track (FT) surgery for joint problems, clinicians pursue the goals of painless, edema-free, stasis-free, and tubeless rehabilitation management after surgery [46]. For post-operative pain management, we should not only adopt the multi-mode analgesia model [47], but also promote the development of multi-disciplinary [48] approaches, especially by adding the high-quality analgesic methods of traditional medicine and developing the combination of traditional Chinese medicine and modern clinical medicine [49].

This clinical study of opposing needling on analgesia and rehabilitation after TKA is aimed at understanding the feasibility of such a procedure. Under the unified conditions of routine operation mode and routine intraoperative and post-operative analgesic dosage and on the premise of baseline equilibrium of the three groups, three interventions will be given according to each patient’s random number: opposing EA, operated side EA, sham EA.

Acupuncture on the affected side is a conventional non-drug therapy for rehabilitation after TKA [20, 50]. Opposing needling means applying acupuncture on the healthy leg (unoperated) of the patient, where acupoints are selected according to corresponding acupoints and meridian of the operated side. Accumulating evidence from clinical and experimental studies revealed that opposing needling exerts protective and facilitative effects on functional rehabilitation, the underlying mechanism of which remains to be further studied [51]. For patients, needling the healthy side after operation is more convenient and more acceptable. In terms of risk, needling the affected side increases the risk of infection after TKA. At the same time, the opposing needling method avoids the disadvantage of inconvenient selections of acupoints distributed at the surgical site.

In addition to the evaluation of the curative effect during hospitalization, good communication and follow-up with patients will be established. Not only will it help patients physically, but also care for patients psychologically. Through the study, we hope to provide clinicians with high-quality evidence of acupuncture for the recovery of pain and function after TKA and provide more ideas for clinicians in perioperative pain management.

## Data Availability

The datasets will be available upon reasonable request after completion of the study.

## Abbreviations

TKA: Total knee arthroplasty
EA: Electroacupuncture
KOA: Knee osteoarthritis
CPSP: Chronic postsurgical pain
SAEs: Severe adverse events
PCA: Patient-controlled analgesic
VAS: Visual analogue scale
CRF: Case report form
ASA: American Society of Anesthesiologists
ST: Stomach meridian of foot*-yangming*
SP: Foot-*taiyin* spleen meridian
GB: Gall bladder channel of foot-*Shaoyang*
HSS: Hospital for special surgery
ROM: Range of motion
COK: Circumference of the knee
HAMA: Hamilton Anxiety Scale
AEs: Adverse events
SPSS: Statistical Package for Social Science
ITT: Intention-to-treat
ANOVA: One-way analysis of variance
FT: Fast-track
